# A Reappraisal of Chemotherapy-Induced Liver Injury in Colorectal Liver Metastases before the Era of Antiangiogenics

**DOI:** 10.1155/2013/314868

**Published:** 2013-03-07

**Authors:** Eric Nguyen-Khac, Céline Lobry, Denis Chatelain, David Fuks, Jean Paul Joly, Marie Brevet, Blaise Tramier, Charlotte Mouly, Vincent Hautefeuille, Bruno Chauffert, Jean Marc Regimbeau

**Affiliations:** ^1^Department of Hepatology, Amiens University Hospital, Place Victor Pauchet, 80054 Amiens Cedex 01, France; ^2^Department of Pathology, Amiens University Hospital, Place Victor Pauchet, 80054 Amiens Cedex 01, France; ^3^Department of Digestive Surgery, Amiens University Hospital, Place Victor Pauchet, 80054 Amiens Cedex 01, France; ^4^Biostatistic Department, Amiens University Hospital, Place Victor Pauchet, 80054 Amiens Cedex 01, France; ^5^Department of Medical Oncology, Amiens University Hospital, Place Victor Pauchet, 80054 Amiens Cedex 01, France

## Abstract

*Background and Aims.* Chemotherapy of colorectal liver metastases can induce hepatotoxicity in noncancerous liver. We describe these lesions and assess risk factors and impacts on postresection morbidity and mortality in naive patients to chemotherapy before the era of bevacizumab. *Methods.* Noncancerous liver tissue lesions were analysed according to tumour, chemotherapy, surgery, and patient characteristics. *Results.* Fifty patients aged 62 ± 9.3 years were included between 2003 and 2007. Thirty-three (66%) received chemotherapy, with Folfox (58%), Folfiri (21%), LV5FU2 (12%), or Xelox (9%) regimens. Hepatotoxicity consisted of 18 (36%) cases of severe sinusoidal dilatation (SD), 13 (26%) portal fibrosis, 7 (14%) perisinusoidal fibrosis (PSF), 6 (12%) nodular regenerative hyperplasia (NRH), 2 (4%) steatosis >30%, zero steatohepatitis, and 16 (32%) surgical hepatitis. PSF was more frequent after chemotherapy (21% versus 0%, *P* = 0.04), especially LV5FU2 (*P* = 0.02). SD was associated with oxaliplatin (54.5% versus 23.5%, *P* = 0.05) and low body mass index (*P* = 0.003). NRH was associated with oxaliplatin (*P* = 0.03) and extensive resection (*P* = 0.04). No impact on mortality and morbidity was observed, apart postoperative elevation of bilirubin levels in case of PSF (*P* = 0.03), longer hospitalization in case of surgical hepatitis (*P* = 0.03), and greater blood loss in case of portal fibrosis (*P* = 0.03). *Conclusions.* Chemotherapy of colorectal liver metastases induces sinusoidal dilatation related to oxaliplatin and perisinusoidal fibrosis related to 5FU, without any impact on postoperative mortality.

## 1. Introduction

Synchronous or metachronous liver metastases (LMs) complicate the course of colorectal cancers (CRCs) in 40% of cases. Surgical resection of LM is the standard treatment, allowing a 5-year survival rate estimated to be between 25 and 44% [1]. Over the past decade, substantial improvement has been obtained in terms of systemic chemotherapy for CLM including perioperative [2] and induction [3] chemotherapies. For patients with initially unresectable disease, induction chemotherapy is offered with a goal of converting these patients to a resectable situation with a 5-year survival rate after resection reaching 35% [1, 3]. However, regardless of its benefit, subsequent toxicity on the nontumorous liver parenchyma has been recently reported in this setting. Various authors have reported increased morbidity and mortality rates after liver resection in patients who received preoperative oxaliplatin or irinotecan-based regimens [4–6]. Several arguments support that irinotecan-based chemotherapy is involved in a histopathologic entity defined as chemotherapy-associated steatohepatitis which manifests as liver steatosis, lobular inflammation, and ballooning of hepatocytes [7–10] that seems to increase both morbidity and mortality after liver resection. Oxaliplatin has been associated with the sinusoidal obstruction syndrome (SOS) and, less frequently, with regenerative nodular hyperplasia [11, 12]. In the context of liver surgery, SOS could increase the risk of intraoperative bleeding [10, 13] and postoperative liver insufficiency [2]. However, the correlation between chemotherapy (type and number of cycles), liver injury (frequency and type of lesions), and clinical outcome (postoperative morbidity and mortality) after liver resection for CLM is currently under debate. In addition, we did not observe that much liver injury after neoadjuvant CT in our experience. Data concerning the effect of antivascular endothelial growth factor (VEGF) and antiepidermal growth factor receptor (EGFR) on nontumoral hepatic parenchyma are more limited [14–17]. Adding bevacizumab to chemotherapy does not increase the injury in non-tumoral hepatic parenchyma: SOS was observed in 27% of patients who received preoperative bevacizumab versus 53% in patients receiving 5-FU and oxaliplatin in a recent series [18]. Therefore, some authors have even stated that anti-VEGF has a protective effect, based on the fact that circulating VEGF is correlated to the severity of SOS [19, 20]. 

The primary objective of this retrospective study was to describe histological lesions of the liver in patients with colorectal cancer treated by neoadjuvant chemotherapy followed by liver resection before the era of bevacizumab in patients naive to chemotherapy. The secondary objectives were to identify factors associated with this liver injury and estimate the impact on postoperative morbidity and mortality. 

## 2. Patients and Methods

### 2.1. Patient's Selection

This was a retrospective analysis of data collected from patients with CLM managed in our Federation in the Amiens University Hospital from February 2003 to July 2007. A prospective database of 485 hepatectomies was used to identify all patients who underwent liver resection for CLM. Among them, 50 patients met the inclusion criteria which were the following: hepatectomy for documented CLM, no underlying chronic liver disease (nonalcoholic, hepatitis B or C virus, or autoimmune chronic liver disease or genetic haemochromatosis), and with sufficient non-tumorous liver parenchyma for pathologic analysis. Among these 50 patients, 33 received preoperative systemic chemotherapy (induction or perioperative CT excluding patients receiving adjuvant CT after primary colorectal resection) within 4 months before hepatectomy (Chemo+) and were compared to the 17 remaining patients who did not receive any chemotherapy (Chemo−). The patients who received bevacizumab were not included. The study was performed in line with the 2000 Declaration of Helsinki. All patients signed an informed consent form.

### 2.2. Preoperative Evaluation

All patients underwent a preoperative evaluation including an abdominal and thoracic CT scan. Patients were considered for hepatectomy if all detected tumors could be removed completely with grossly negative surgical margins and a safe liver remnant volume. In selected patients in whom the amount of future remnant liver was considered insufficient (less than 30% of total liver volume) [21, 22], a preoperative portal vein embolization was performed [23]. Biologic data assessed before surgery were the following: platelet count, serum creatinine level, serum aspartate aminotransferase level (AST), serum alanine aminotransferase level (ALT), gamma glutamyl transferase level (*γ*GT), alkalin phosphatase level (AP), serum total bilirubin level (Bili), and prothrombin time (PT).

### 2.3. Indication and Regimens of Systemic Chemotherapy in Chemo+ Group

In resectable patients (*n* = 5), indication for preoperative chemotherapy were to downsize the tumors preoperatively in view of a function-sparing resection or to ensure negative margins (*n* = 3) and to assess tumor's response to chemotherapy (*n* = 2). The patients with nonresectable CLM at presentation (*n* = 28) received “induction” chemotherapy which aimed at downsizing the CLM to switch the patients from a “non-resectable” status to a “resectable” status. 

### 2.4. Surgical Technique

Liver resections were performed at least 4 weeks after the last course of chemotherapy in the Chemo+ group. During operation, a thorough exploration of the abdomen and the liver (intraoperative ultrasound) was carried out to rule out any contraindication to liver resection. Vascular clamping was not performed routinely, but if necessary resections were performed preferentially under intermittent portal triad clamping. Liver resection was achieved with a macroscopic tumor-free margin of 1 cm or larger whenever possible. Major hepatectomies were defined as the resection of three or more segments. Liver resection was performed by the same experienced liver surgeon (JMR). 

### 2.5. Intraoperative and Postoperative Course

Intraoperative appearance of the liver, blood loss, use of vasopressive drugs, and blood requirement were recorded. Intra- and postoperative transfusions were taken into account. Mortality was defined as death occurring within 90 days after surgery, and morbidity was defined as a complication occurring during the hospital stay. Complications were stratified in accordance with Dindo's classification [22]: grade I was complications that induce any deviation from the normal postoperative course, grade II was complications that require pharmacological treatment, grade III was complications that require surgical, endoscopic, or radiological intervention, grade IV was life-threatening complications that require intermediate or intensive care unit management, and grade V was complications that result in the death of the patient. Liver dysfunction was defined as follows: ascites (volume > 500 mL per day) and/or PT < 50% on day 5 and/or Bilirubin > 50 *μ*mol/L on postoperative day 5 and/or PT < 30% at any time [22]. The combination of a PT < 50% and a Bilirubin >50 *µ*mol/L on postoperative day 5 was defined as liver failure [24, 25].

As for preoperative management, adjuvant chemotherapy was decided on by a multidisciplinary committee that included oncologists, pathologists, gastroenterologists, radiologists, and surgeons.

### 2.6. Pathologic Analysis

All slides, which were originally prepared from formalin-fixed and paraffin-embedded tissues, were reviewed. Representative slides of non-tumorous hepatic tissue located as far as possible from the tumor were selected for the study. The morphological analyses were performed using slides stained with hematoxylin and eosin, Masson trichrome, and reticulin stain. The slides were examined by a single pathologist with hepatobiliary expertise (DC), who was unaware of the clinical data. *Hepatic steatosis* was classified into 3 grades: less than or equal to 30%, between 30% and 60%, and greater than 60% [26]. *Steatohepatitis* was evaluated according to the semiquantitative score of Kleiner et al. [27] and the NASH activity score (NAS) obtained by the addition of the steatosis (0 ≤ 5%, 1 = 5–33%, 2 = 33–66%, and 3 ≥ 66%), lobular inflammation (0 = no site, 1 ≤ 2 sites, 2 = two to four sites, and 3 ≥ four sites per ×200 field), and hepatocyte ballooning (0 = absent, 1 = several ballooned hepatocytes, 2 = numerous ballooned hepatocytes, or predominant hepatocyte ballooning) scores. A NAS score ≥ 5 was in favour of steatohepatitis, and a score less than 3 excluded steatohepatitis [27]. Sections were examined for the presence of vascular lesions such as *sinusoidal dilatation* (SD) classified into grade I (minimal centrilobular dilatation), II (dilatation occupying 2/3 of the lobule), and III (dilatation occupying all of the lobule) [11]. The presence of *nodular regenerative hyperplasia* was investigated by specific reticulin stain. *Perisinusoidal fibrosis* (PSF) was classified as minimal, moderate, or severe [28]. *Portal fibrosis* was estimated according to the Metavir score: absent (F0), portal fibrosis without septa (F1), portal fibrosis with several septa (F2), numerous septa without cirrhosis (F3), and cirrhosis (F4) [29]. Finally, lesions secondary to intraoperative manipulation of the surgical specimen were defined by periportal or centrilobular hepatocyte necrosis with polymorphonuclear neutrophile infiltrate [13] and were called “*surgical hepatitis*”.

### 2.7. Studied Criteria

Demographic data (age, gender, body mass index, and ASA score (American Society of Anesthesiologists)), associated comorbidities (smoking, diabetes mellitus, arterial hypertension, and hypercholesterolaemia), pathological variables (number and size of CLM, pTNM stage), chemotherapy characteristics (type of chemotherapy, number of courses, dose and duration of chemotherapy, and interval between the end of chemotherapy and liver surgery), surgical modalities (extension of resection, vascular clamping, preoperative portal embolization, macroscopic appearance of the liver, blood loss, number of units of packed cells transfused, and operating time), and postoperative outcomes (mortality, morbidity, and length of stay) were recorded. The laboratory assessment prior to any chemotherapy, then before and after liver surgery, including transaminases, gammaglutamyl transferase, alkaline phosphatase, total serum bilirubin, prothrombin time, and complete blood count and platelets were also recorded. 

### 2.8. Statistical Analysis

All results are expressed as the mean ± standard deviation, or median and range. Statistical comparisons were performed by Mann-Whitney, Wilcoxon Kruskal-Wallis and Student's *t* tests for continuous variables, Fisher's exact test for binary variables and Pearson's Chi-square test for ordinal variables. A *P* value < 0.05 was considered to be significant. All statistical analyses were performed using SPSS 18.0 (SPSS, Inc., Chicago, IL). 

## 3. Results

### 3.1. Patients

**Figure 1:**
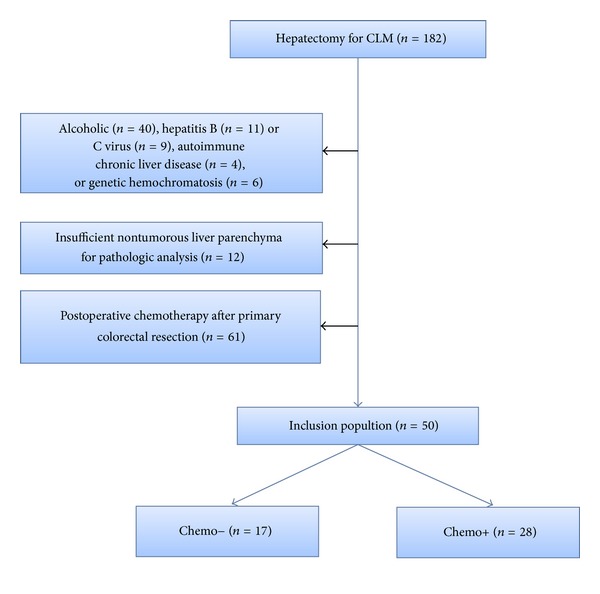
Flow chart of the study.

From 2003 to 2007, a total of 182 patients underwent hepatectomy for CLM in our department. Among these 182 patients, 111 (61%) received neoadjuvant chemotherapy. Sixty-one patients had adjuvant CT after primary colorectal resection and were not analysed (Figure 1). Fifty patients satisfying the inclusion criteria were analysed. There were 34 males and 16 females with a mean age of 62 years (range: 36–82 years). Thirty-three (66%) patients had a primary colonic cancer. CLM was synchronous in 62% of patients. The mean number of metastases was 2.3 (range: 1–7) with a mean diameter of 55.3 mm (range: 47–190). Preoperative characteristics of these patients are summarized in Table 1. In the Chemo+ group, 28 had initially nonresectable CLM, and the remaining 5 patients with resectable CLM received neoadjuvant chemotherapy. In the Chemo− group, 17 patients had primary colorectal tumor with synchronous resectable CLM.

### 3.2. Regimens of Systemic Chemotherapy in Chemo+ Group

Details of CT are reported in Table 2. The mean number of cycles of CT was 9.5 ± 5.5 (range 3–27). Twelve (36%) patients received more than six cycles. The mean time interval between administration of CT and surgery was 25.2 ± 17 days (range 15–41).

### 3.3. Surgical Procedures

Fifteen (30%) patients underwent major hepatectomy (after preoperative portal vein embolization *n* = 3), and 15 (30%) required vascular clamping. The mean operating time was 334 minutes (range 140–600), and 5 (10%) had intraoperative transfusion. Intra-operative liver appearance was normal in 30 (60%) patients, steatotic in 12 (24%), and congested in 8 (16%) patients. There was no difference between Chemo+ and Chemo− groups.

### 3.4. Postoperative Outcomes

Postoperative mortality was 4% (*n* = 2), and overall morbidity was 40% (*n* = 20). Dindo's III-IV complications included 6 patients. No liver or renal failure was reported. There was postoperative ascites in one patient who had major hepatectomy. The median length of hospitalization was 15.2 days (range 5–48).

### 3.5. Description of Histological Lesions of the Liver

Histological examination of non-tumorous liver parenchyma demonstrated hepatic steatosis ≤ 30% in 40 (80%) and steatosis >30% in 2 (4%) patients. No patients had NASH (median NAS score of 2 (range: 0–4)). Six (25%), 10 (42%), and 8 (33%) patients had grade I, II, and III sinusoidal dilatation, respectively. Nodular regenerative hyperplasia was observed in 6 (12%) patients. Perisinusoidal fibrosis was minimal in 9 (18%) and moderate to severe in 7 (14%) patients. Thirty seven patients had F0 fibrosis and 13 (26%) had more than F2 portal fibrosis. “Surgical hepatitis” lesions were described in 16 (33%) cases. Only perisinusoidal fibrosis was significantly higher in Chemo+ group than in Chemo− group (*P* = 0.04) (Table 3).

When the surgeon intraoperatively observed macroscopic steatosis, the histological steatosis was 21 ± 42%, whereas it was 9.6 ± 10.3% when the macroscopic appearance was normal (*P* = 0.02). On the contrary, the macroscopic appearance of the liver was not associated with presence of sinusoidal dilatation on the specimen (*P* = 0.08).

### 3.6. Association between Hepatic Lesions and the Various Chemotherapy Protocols

Only LV5FU2 chemotherapy was significantly associated with perisinusoidal fibrosis, compared to patients not receiving chemotherapy (*P* = 0.02) (Table 4). Patients with PSF received an average of 12.8 courses of 5FU-based chemotherapy versus 8.8 courses for patients without PSF, but the difference was not significant (*P* = 0.15).

Grade II and III sinusoidal dilatation was present in 54.5% of patients who had received oxaliplatin-based chemotherapy (Folfox or Xelox) versus 23.5% in Chemo− patients (*P* = 0.05), while no difference was observed with the other chemotherapy protocols used. The mean number of courses of oxaliplatin-based chemotherapy was 8.8 in the group of patients with sinusoidal dilatation versus 9 in the absence of sinusoidal dilatation (*P* = NS). Five (22.7%) of the 22 patients who had received oxaliplatin-based chemotherapy (Folfox or Xelox) presented features of NRH versus only 1/17 (5.9%) patients in Chemo− group, but this difference was not significant (*P* = 0.20). However, patients with NRH received more courses of oxaliplatin-based chemotherapy than patients without NRH (10.2 ± 4 courses versus 7.6 ± 3.7 courses, *P* = 0.03).

Hepatic steatosis greater than 30% and significant portal fibrosis (*F* ≥ 2) were not associated with chemotherapy. Surgical hepatitis lesions were not associated with chemotherapy, but appeared to be more frequent in patients who had received Folfox (42.1%) or Folfiri (42.9%), versus 29.4% in the absence of chemotherapy, but the differences were not statistically significant. 

### 3.7. Association between Hepatic Lesions and other Risk Factors

**Figure 2:**
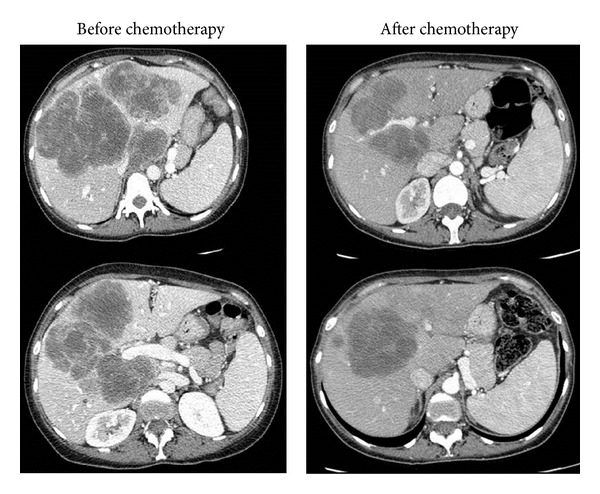
Example of a 41-year-old patient who developed HNR with portal hypertension after induction chemotherapy (FOLFOX, IV 12 cycles). An increase of the size of the spleen and an apparition of portal hypertension was observed after chemotherapy.

Among the demographic factors related to the patient (age, gender, BMI, smoking, hypercholesterolaemia, and diabetes mellitus), the primary tumor, the CLM (number and size of LM), and timing of chemotherapy (duration and interval between end of chemotherapy and surgery), only diabetes mellitus was significantly associated with hepatic steatosis greater than 30% (*P* = 0.02). BMI was significantly lower in patients with sinusoidal dilatation compared to patients without sinusoidal dilatation (25.6 ± 4.7 versus 29 ± 3.7, *P* = 0.003). Perisinusoidal fibrosis, NRH and portal fibrosis were not associated with any of these factors (Figure 2). Finally, patients with surgical hepatitis lesions had a shorter interval between the end of chemotherapy and surgery than patients without this type of lesion (59 ± 29.8 days versus 191 ± 212.2 days, *P* = 0.019).

### 3.8. Consequences of Histological Lesions on Surgery, Liver Function Tests, Mean Length of Stay, and Postoperative Outcomes

Intra-operative blood loss was increased in the presence of portal fibrosis *F* ≥ 2, compared to patients with *F*0-*F*1 fibrosis (1,045 ± 880 versus 541 ± 652 mL, *P* = 0.03). Major hepatectomy was associated with a higher incidence of NRH (*P* = 0.04). The only significant postoperative modification of liver function tests was a more marked elevation of serum bilirubin levels in the presence of PSF (bilirubin increased by 18.6 ± 28.3 versus 13.7 ± 26.4 *μ*mol/L, *P* = 0.05).

The mean length of hospital stay was not influenced by steatosis (*P* = 0.99), perisinusoidal fibrosis (*P* = 0.16), sinusoidal dilatation (*P* = 0.56), NRH (*P* = 0.80), or portal fibrosis *F*2 (*P* = 0.20), but the mean length of hospital stay was significantly longer in case of surgical hepatitis (18 ± 10.7 versus 14 ± 10.3 days, *P* = 0.03). Among the 50 patients, postoperative morbidity was not modified by hepatic lesions: steatosis (*P* = 0.99), PSF (*P* = 0.82), sinusoidal dilatation (*P* = 0.69), surgical hepatitis lesions (*P* = 0.36), or NRH (*P* = 0.35) on the Mann-Whitney test. No difference in postoperative morbidity was observed between Chemo+ and Chemo- groups. Among Chemo+ patients, there was no difference in postoperative morbidity in patients who presented at least one liver injury compared to patients with no liver injury (median Dindo's score: 1.8 ± 1.2 versus 1.14 ± 0.37, *P* = 0.45). We did not find any difference of postoperative morbidity with or without PSF (*P* = 0.48) or sinusoidal dilatation (*P* = 0.056). We did not demonstrate any impact of histological lesions on postoperative mortality. Two patients died during the postoperative period with no signs of vascular lesions (SD, NRH, or PSF) or portal fibrosis *F* ≥ 2. Both patients presented signs of surgical hepatitis with steatosis scores of 30% and 10%, respectively.

### 3.9. Postoperative Outcomes and the Various Chemotherapy Protocols

Post-operative mortality was 4% (*n* = 2) with no correlation with the number of courses of oxaliplatin-based chemotherapy (*P* = 0.51) and irinotecan-based chemotherapy (*P* = 0.9).

Overall morbidity was 40% (*n* = 20) included 6 patients with Dindo's III-IV. There was no association between the overall morbidity and the number of courses of chemotherapy included oxaliplatin-based chemotherapy and irinotecan-based chemotherapy (*P* > 0.05). The number of courses >6 (or >8) for oxaliplatin-based chemotherapy and the number of courses >6 (or >8) for irinotecan were not associated with a higher incidence of morbidity. Finally, the number of course of chemotherapy protocol did not influence postoperative outcomes. 

## 4. Discussion

Over the past decade, preoperative chemotherapy is being increasingly used before hepatic resection for colorectal liver metastases [3, 30]. Several arguments support its use in selected patients: [3] by downsizing the tumors preoperatively, it may increase the rate of curative resection with negative margin, [31] some patients with unresectable disease at presentation may become eligible for hepatic resection, [30] good responders may be identified preoperatively, and [2] response to chemotherapy may be a good evaluation of tumors biologic aggressiveness as those who progress under chemotherapy may not benefit from resection [10, 32]. Hepatotoxicity induced by the chemotherapy protocols used in colorectal cancer is an emerging problem and raises the question of whether this hepatotoxicity may interfere with the results of management of CLM in terms of postoperative morbidity and mortality. The present study population was comparable to those of other published series in terms of age, gender, BMI, colorectal tumour characteristics, mean number of CLM, and history of chemotherapy prior to surgery [7, 11, 13, 33]. However, this series included all types of neoadjuvant and adjuvant chemotherapy except bevacizumab and patients who had postoperative chemotherapy after primary tumor resection, regardless of the date of administration in relation to liver surgery, while other studies excluded from the Chemo+ group patients who had received chemotherapy more than 6 months before surgery [13] and sometimes even more than 2 months before surgery [34]. The mean interval between the end of chemotherapy and liver surgery was longer in our series than in other studies [7, 11], which could influence the prevalence of reversible lesions such as steatosis. Finally, the nonrandomized retrospective nature of our study is limitations common to all recently published studies [7, 9, 11, 13, 34, 35], apart from a European prospective study [36].

Perisinusoidal fibrosis and sinusoidal dilatation were observed in 7 (14%) and 18 (36%) patients, respectively, and were significantly correlated with previous chemotherapy. PSF was significantly more frequent in the group of patients that had received chemotherapy (*P* = 0.04), particularly 5FU monotherapy (*P* = 0.02). Patients with PSF had received a greater number of courses of 5FU-based chemotherapy than patients without PSF, but the difference was not significant. This result must be interpreted cautiously in view of the small sample size. PSF has been reported in only one other study [11] and was interpreted by the authors to be a late consequence of sinusoidal dilatation. PSF can be either isolated or associated with sinusoidal dilatation and obstruction [37]. It can also be due to nonalcoholic steatohepatitis [26, 38], but these two risk factors were not associated with PSF in our study. To our knowledge, no previous study has reported an association between PSF and 5FU. This hypothetical association, therefore, needs to be confirmed. PSF did not induce any postoperative morbidity or mortality and did not even increase the mean hospital length of stay, although a more marked elevation of total serum bilirubin was observed during the postoperative period in patients with PSF. Elevation of serum bilirubin could be due to capillarization of sinusoids, secondary to fibrosis, which interferes with metabolic exchanges.

This study also confirms the existence of sinusoidal dilatation in noncancerous liver with a prevalence of 36%. This rate was 54.5% for patients who had received oxaliplatin-based chemotherapy versus 23.5% for patients without chemotherapy (*P* = 0.05). Previous studies have reported an association between sinusoidal dilatations and chemotherapy and no chemotherapy [13, 34], not confirmed in the present study, and between the use of oxaliplatin [7, 11] exclusively in severe grade II and III lesions, as demonstrated in the present study. The reported prevalence of sinusoidal dilatations is between 8% [7] and 50% [11, 34] among patients receiving neoadjuvant chemotherapy, increasing to 19% and 79% for patients treated with oxaliplatin [11, 36]. Rubbia-Brandt et al. were the first to report the development of sinusoidal dilatation after chemotherapy and proposed the following hypothesis: the initially damaged endothelial cells induce activation of stellate cells, leading to fibrosis and aggregation of erythrocytes and cytoplasmic blebs in the perisinusoidal space, which results in obstruction of the junction between sinusoids and centrilobular venules [11]. Apart from chemotherapy, sinusoidal dilatation was also significantly more frequent in patients with a lower BMI (*P* = 0.003), but this was not confirmed by another study [36]. This result could suggest differences in the metabolism of chemotherapeutic agents related to BMI. No other risk factor was associated with the presence of sinusoidal dilatation in our study: neither duration or number of courses of chemotherapy, the interval between the end of treatment and surgery, the extent of liver resection, nor the use of vascular clamping. Similarly, other authors also did not report any correlation between sinusoidal dilatations and the duration of chemotherapy [7, 13] or the cumulative dose of oxaliplatin [11]. Only Farges et al. reported a tendency to a greater number of severe sinusoidal dilatation in the case of intraoperative vascular clamping, but the difference was not significant (*P* = 0.09) [33]. As reported in other series, sinusoidal dilatation did not have any impact on the mean length of hospital stay [34], transfusion [13, 34], or postoperative morbidity and mortality [7, 34, 36]. 

None of the other hepatic lesions observed were associated with any of the chemotherapy protocols used. The incidence of 12% of NRH in this study was comparable to the rates reported in the literature [11, 13, 34]. Patients with NRH had received significantly more courses of oxaliplatin-based chemotherapy (*P* = 0.03). This confirms the results of Rubbia-Brandt et al. [11], whom described NRH as secondary to the use of oxaliplatin, frequently associated with sinusoidal dilatation. This is coherent with our results concerning sinusoidal dilatations and oxaliplatin. The presence of NRH did not influence the postoperative course, transfusion requirements, or survival rate, as reported by Farges et al. [33]. NRH lesions, therefore, appeared to have an identical risk profile to that of sinusoidal dilatation, as they also belong to the spectrum of vascular lesions, suggesting a continuum of these oxaliplatin-induced lesions [38, 39].

Hepatic steatosis in non-tumoral liver parenchyma was frequent in our population, observed in almost 80% of cases, despite the fact that our patients had a slightly high BMI. However, steatosis >30% was much rarer, observed in only 8% of cases, while other studies have reported frequencies between 9% and 20% [7, 11, 13] with no significant association with chemotherapy [11, 13, 34, 36], except for the series by Pawlik et al. in which hepatic steatosis was significantly correlated with the use of irinotecan [35]. As in the setting of liver transplantation, steatosis >30% could increase the postoperative morbidity after resection of CLM [40, 41], but this remains controversial [34, 42]. No conclusions can be drawn from the present study due to the low rate of steatosis >30% in this population. While steatohepatitis has been significantly associated with irinotecan use, inducing increased mortality on the 90th postoperative day from liver failure (*P* = 0.01) [17], no case of this complication was observed in our study based on the use of the NAS score proposed by Kleiner et al. Similarly, Fernandez et al. [9], based on a small sample size (*n* = 37), noted that chemotherapy, with no distinction between oxaliplatin or irinotecan, was a risk factor for steatohepatitis, independent of BMI, compared to patients who had not received chemotherapy or who received 5FU alone (*P* = 0.003). However, no consensus has been reached concerning the histological criteria for the diagnosis of steatohepatitis. Vauthey et al. [7] used a NAS score > 4, while Fernandez et al. [9] used the inflammatory activity score of Brunt et al. Finally, other authors did not report any association between steatohepatitis and oxaliplatin or increased morbidity or mortality [34–36]. Data concerning steatosis and steatohepatitis, therefore, vary from study to study related to population differences in terms of metabolic risk factors, interval after the end of chemotherapy, or even the use of irinotecan.

Portal fibrosis, *F* ≥ 2, was not associated with chemotherapy in this study, confirming several previous reports [13, 34]. It was not related to extended liver resection or the operating time. However, an increased blood loss (*P* = 0.03) and a tendency to greater transfusion requirement (*P* = 0.09) were observed in the presence of *F* ≥ 2, not confirmed by other series [13, 33]. 

Lastly, the pathophysiology of “surgical hepatitis” remains poorly defined, but could correspond to zones of infarction related to ischaemia. When major resections with total vascular exclusion were performed, surgical hepatitis lesions were observed in 97% of cases in one study [34]. Thirty-three percent of our patients presented surgical hepatitis, but it was not correlated with major resection, vascular clamping, operating time, or blood loss; postoperative liver function tests and postoperative course did not differ from those of the other patients, apart from a significantly longer length of hospital stay (*P* = 0.03). Furthermore, the 2 patients who died during the postoperative period presented surgical hepatitis and no other histological lesion apart from steatosis scores of 10 and 30%. Surgical hepatitis was not associated with chemotherapy in our study, as in the series by Karoui et al. [34], but a correlation with chemotherapy was observed in another series [13]. However, surgical hepatitis was significantly more frequent in our study when the interval between the end of chemotherapy and surgery was shorter (*P* = 0.019), suggesting a possible impact of chemotherapy on the susceptibility of non-tumoral liver parenchyma to ischaemia.

Regarding duration of chemotherapy exposure prior to resection, Karoui et al. reported that patients receiving systemic chemotherapy (mostly oxaliplatin) had a significantly higher rate of sinusoidal dilatation (49 versus 13.6%, *P* = 0.005) and postoperative complications (38 versus 13.5%, *P* = 0.03) compared with controls. This correlated with the number of chemotherapy cycles administered. Patients who received >6 cycles had considerably higher postoperative complications, as compared with those treated with <6 cycles (54 versus 19%, *P* = 0.047) [34]. Moreover, in the study of Kneuertz et al. [43], postoperative liver failure was observed in five patients who received more than ten cycles of chemotherapy. However, in our study we found that a number of courses >6 for oxaliplatin-based chemotherapy and a number of courses >8 for irinotecan were not associated with a higher incidence of morbidity.

In conclusion, hepatic sinusoidal dilatation is probably the most frequently reported and demonstrated chemo-induced liver injury in patients receiving chemotherapy for CLM. Oxaliplatin is usually responsible [7, 11, 35]. Other lesions such as steatosis [7, 11, 13, 34, 35] have been less clearly demonstrated, while in 3 studies [7, 9, 35] steatohepatitis was associated with the use of irinotecan. The impact of all of these lesions on postoperative morbidity and mortality remains controversial [7, 13, 34, 35]. At the present time, the benefit-risk balance remains in favour of chemotherapy for colorectal LM. However, a short window after the last course of chemotherapy to perform the liver resection, giving time for regeneration the non-tumorous liver parenchyma would be considered, but not too long time for tumor escaping.

## Figures and Tables

**Table 1:** Baseline characteristics of the 50 included patients.

	Parameters	Total *n* (%)	Chemo+ (*n* = 33)	Chemo− (*n* = 17)	*P*
Demography	Age (years)	62 ± 9.3	60	63	NS
	Male gender	34 (68)	22 (66)	12 (71)	NS
	ASA score (I/II/III)	8 (16)/26 (52)/16 (32)	7/15/11	1/11/5	NS
	BMI	28 ± 4.7	27	28	NS

Comorbidities	Hypertension	21 (42)	14 (44)	7 (41)	NS
	Diabetes mellitus	4 (8)	3 (9)	1 (6)	NS
	Smoking	16 (32)	11 (33)	5 (29)	NS
	Hyperlipidemia	14 (28)	10 (30)	4 (24)	NS

Liver tests	AST (IU/L)	30.5 ± 15.2	29	33	NS
	ALT (IU/L)	31.2 ± 19	24	38	NS
	*γ*-Glutamyl transferase (IU/L)	86 ± 97	82	90	NS
	Alkaline phosphatase (IU/L)	110 ± 47.7	110	108	NS
	Prothrombin time (%)	89.6 ± 2.6	90.6	88.4	NS
	Total bilirubin (*µ*mol/L)	9.2 ± 5.6	8.4	10.0	NS
	Platelets count (×10^3^/mm^3^)	247 ± 111	220	251	NS

ASA: anesthesiologist score association.

BMI: body mass index.

NS: not significant.

**Table 2:** Characteristics of chemotherapy in the Chemo+ group (*n* = 33).

	Parameters	*n* (%) Mean ± SD
Type of CT	LV5-FU2 *n* (%)	4 (12)
	Folfox *n* (%)	19 (58)
	Xelox *n* (%)	3 (9)
	Folfiri *n* (%)	7 (21)

Number of courses	Fluorouracil	10.4 ± 5.7
	Fluorouracil plus irinotecan	8 ± 6.7
	Fluorouracil plus oxaliplatin	8.2 ± 3.8

Cumulative dose	Fluorouracil (mg)	51580 ± 29520
	Irinotecan (mg)	2767 ± 2250
	Oxaliplatin (mg)	1250 ± 626

**Table 3:** Histological lesions of the non-tumorous liver parenchyma.

Histological lesions of the non-tumorous liver parenchyma	All	Chemo+ (*n* = 33) *n* (%)	Chemo− (*n* = 17) *n* (%)	*P*
Steatosis ≤ 30%	40 (80)	29 (88)	11 (65)	NS
Steatosis > 30%	2 (4)	1 (3)	1 (6)	NS
Steatohepatitis (NASH), NAS ≥ 5	0 (0)	0 (0)	0 (0)	—
Grade II and III sinusoidal dilatation	18 (36)	14 (42)	4 (24)	NS
Nodular regenerative hyperplasia (NRH)	6 (12)	5 (15)	1 (6)	NS
Moderate to severe perisinusoidal fibrosis	7 (14)	7 (21)	0 (0)	0.04
Portal fibrosis ≥ F2	13 (26)	10 (31)	3 (18)	NS
Mild/severe postoperative lesions	9 (18)/16 (32)	7 (21)/12 (36)	2 (12)/4 (24)	NS/NS

**Table 4:** Characteristics of liver impairment according to the type of chemotherapy.

Chemotherapy	Steatosis > 30%			Portal fibrosis ≥ 2			Perisinusoidal fibrosis			Sinusoidal dilatation			NRH		
	(*n* = 2)			(*n* = 13)			(*n* = 7)			(*n* = 18)			(*n* = 6)		
	Chemo+	Chemo−	*P*	Chemo+	Chemo−	*P*	Chemo+	Chemo−	*P*	Chemo+	Chemo−	*P*	Chemo+	Chemo−	*P*
LV5FU2	0/4 (0)	1/17 (6)	1	2/4 (50)	3/17 (17.6)	0.22	2/4 (50)	0/17 (0)	0.02	1/4 (25)	4/17 (23.5)	1	0/4 (0)	1/17 (5.9)	1
Folfox	1/19 (5.3)	1/17 (6)	1	5/18 (27.8)	3/17 (17.6)	0.69	4/19 (21.1)	0/17 (0)	0.10	10/19 (52.6)	4/17 (23.5)	0.07	4/19 (21)	1/17 (5.9)	0.34
Xelox	0/3 (0)	1/17 (6)	1	0/3 (0)	3/17 (17.6)	1	0/3 (0)	0/17 (0)	1	2/3 (66.6)	4/17 (23.5)	0.20	1/3 (33.3)	1/17 (5.9)	0.28
Folfox and Xelox	1/22 (4.5)	1/17 (6)	1	5/21 (24)	3/17 (17.6)	0.70	4/22 (18.2)	0/17 (0)	0.11	12/22 (54.5)	4/17 (23.5)	0.05	5/22 (22.7)	1/17 (5.9)	0.20
Folfiri	0/7 (0)	1/17 (6)	1	2/6 (33.3)	3/17 (17.6)	0.57	2/7 (28.6)	0/17 (0)	0.07	2/7 (28.6)	4/17 (23.5)	1	1/7 (14.3)	1/17 (5.9)	0.50

## Abbreviations

CT: Chemotherapy
CLM: Colorectal liver metastases
NRH: Nodular regenerative hyperplasia.
